# Functional Diseases of the Eye—Hypermetropia

**Published:** 1873-05

**Authors:** Swan M. Burnett

**Affiliations:** Knoxville, Tennessee


### SOUTHERN MEDICAL RECORD.

VOL. III.—MAY, 1873.—NO. 5.

---

### PART I.

---

### ORIGINAL COMMUNICATIONS.

---

### FUNCTIONAL DISEASES OF THE EYE—HYPERMETROPIA.

BY SWAN M. BURNETT, M.D., KNOXVILLE, TENNESSEE.

The functional affection of the eye that we shall next consider is *Hypermetropia*, (*hyper*, beyond, *metros*, measure), which, as its name indicates, is vision beyond the normal limits. It is commonly known as *far-sightedness*. For a long time it was confounded with Presbyopia or old eyes, but recent investigations have shown that the two affections depend upon widely-differing pathological conditions. We will defer the consideration of these differences in detail until we come to consider the latter-named affection; suffice it here to state, that while Presbyopia is an anomaly of the Accommodation, hypermetropia is an anomaly of Refraction.

It is a condition exactly opposite to M. While in M. we had parallel rays brought to a focus *before* the retina during rest of Accommodation, in H., under the same condition, we have them focused *behind* the retina.

It was early discovered that convex glasses would improve the vision of far-sighted persons, and upon this fact were based many theories concerning the affection. The reasoning was in the most instances good, the only difficulty being that they did not start upon the right premises, and neglected to verify their hypotheses by a further examination into the facts. They were correct in referring the trouble to a want of proper focusing of the rays upon the retina, and in supposing that the focus was *behind* it—the benefit from the convex glasses would teach them this; but they were universally in error in assigning a cause for it. By some it was referred to a lessening in the curvature of the lens; by others—and these were in the majority—to a flattening of the cornea. Either of these conditions, had it been present, would have indeed produced the hypermetropic condition; but, to the detriment of their theories, they neglected to *prove* that such was the case.

In our article on M., we mentioned that Donders had invented an instrument for measuring, with great exactness, the curvature of the lens and cornea. Here, then, was a test—a crucial test—for their theories. It was applied, and their flimsy fabrics crumbled.

In H., Donders has proved, beyond doubt, that the curvature of the cornea is not only not less, but is *greater* than in the normal eye, and the curvature of the lens' surfaces he found to differ but very slightly from the normal, or from the myopic eye. The situation of the principal focus behind the retina must, then, be accounted for in some more satisfactory manner. He was not long in finding a solution of the difficulty, and even succeeded in demonstrating that *H. depends upon a shortening of the antero-posterior diameter of the globe.* This is a condition diametrically opposite to that we have seen is present in M., and it is demonstrable by measurement after extirpation, and by the ophthalmoscope.

### VARIETIES.

H. is divided into three forms—the *Absolute, Relative,* and *Facultative*—and it is important that, in every case that comes under our observation, it be determined to which class it belongs. Our therapeutic measures, as we shall see, will be often influenced by the form the affection assumes.

In the *Absolute* form, the patient is not able, with all the Accommodation at his command, to bring parallel rays to a focus upon the retina, and consequently, to him, even distant objects are indistinct. In the *Relative* form, by sufficient Accommodation, distinct images may be formed on the retina; but, owing to the intimate relation between the Accommodation and convergence of the visual axes, these images can be obtained only when the binocular nearest point is nearer to the eyes than the object. This will, perhaps, require some further elucidation. Such an intimacy exists between A. and convergence that, for a certain amount of A. put forth, there will be a certain convergence of the visual axes, and *vice versa*. In the normal eye, however, this is so adjusted that when the visual axes cross at a particular point, just that amount of A. is called into requisition as shall cause a well-defined image of that point upon corresponding parts of the two retinæ, and thus binocular vision is made possible. It will be readily seen that if this relation is in any way disturbed, that the image either will not fall upon corresponding percipient retinal elements, or, if it does, will not be clear and well-defined (because the eyes are not accommodated for it).

Such a disturbance does exist in Relative H. The A. is so much in excess of convergence that, in order to have a distinct image, the point of crossing of the visual axes must fall between the object and the eyes. The image, then, does not fall upon corresponding percipient elements of the retina, and diplopia, or double vision, arises. Hence, in this form of H., the patient has either to submit to much obscurity of vision, or give up binocular vision, fixing only with one eye.

This law of relative Accommodation, though founded upon correct theory and supported by numerous facts, is still sometimes counteracted by another law stronger, viz.: the desire for binocular vision. When an excessive amount of A. is called for, if it is not too large, the Hypermetrope is enabled to fix and accommodate for the same object; counteracting the tendency to squint by an extra tension on the part of the external recti muscles. The limits of this power, however, are quite small, still it is possible, and when it is brought into requisition, we have *Facultative* H., in which, during parallelism of the visual axes, a small amount of A. can be used, and parallel rays thus brought to a focus upon the retina.

As the Accommodation gives way with age, this becomes impossible, and the Facultative then runs into the Relative form, as the Relative, under the same circumstances, glides into the Absolute.

There is another fact of great importance to be noted, viz.: that H. may in part be *latent.* On making the trial by glasses, even very weak convex glasses, instead of improving V., will often make objects more indistinct. This is most frequently observed in very young persons, whose Accommodation is active. The activity of their A. enables them to overcome a portion, or it may be all, of their H., and thus in some instances there is danger of running into the error of supposing that there is no H. at all. This excess of A., after a time, becomes a normal condition, and its relation to the convergence becomes in a degree fixed, and it is only by entirely neutralizing the A. by atropine that we are aware of the exact state of the Refraction. In cases where we have reason to suspect H., and positive glasses give no improvement, the A. should always be paralyzed. This power of suppressing H. is so strong in some, that an H. of 1-6 is often rendered latent, and frequently in Hm. (manifest Hypermetropia) of 1-10 or 1-12, on paralysis of A., we find Ht. (total Hypermetropia)=1-5 or 1-6. Of course, this is only in young persons with very active A. It is in just such cases that that very painful and troublesome complication of H. Asthenopia arises, and which, prior to the discoveries of Donders, could only be relieved by absolute abstinence from work, and, what is more melancholy, often kept the patient in constant fear of finally losing his vision altogether—a fear of which his physician was powerless to relieve him. Imagine the joy of these persons when they find that their disease is only functional, and that, by the proper use of spectacles, they will be able to use their eyes for all purposes with ease and comfort!

### *SYMPTOMS.*

The most characteristic symptom is the inability to see near objects, as the letters of a book, with distinctness, or if they are seen distinctly, soon run into each other, and become confused and blurred. The use of the eyes for near work for any length of time is rarely unattended with pain. After a few moments' rest, however, work can be resumed with comparative comfort, to be again followed by the pain and dimness, necessitating another rest.

The following case from my note-book will perhaps give a better idea of the symptoms than any mere description:

W. M., æt. 11, finds that in studying his lesson, after a time the letters and lines run into each other, and become indistinguishable. He has pains over his eyes, and is forced to close the lids to get relief. By turning his head to one side, and looking only with one eye, he is able to read better and for a longer time. By artificial light he is scarcely able to read at all. I suspect H. at once, and on examination find Hm.=1-13. I give him + 1-13, and with them he is able to read his lesson with ease, and as well by artificial light as by day.

But in high degrees of H., especially if the patient is somewhat advanced in life, even distant objects are not seen distinctly; and often, in order to obtain a good view of near objects, they are brought to within two or three inches of the eye. This leads frequently to the supposition of a high degree of M. The following is a case in point:

Mrs. S., æt. forty-three, even as a girl, had trouble in seeing near objects. She was often unable to thread her needle, particularly after sewing for some time, and her head frequently ached after work. She began to use convex glasses early (at about thirty) for near work, but soon found that she could not see well at a distance without them. Soon, even with their use, near work became painful, and she was in great fears for her vision. She had continued in this state of mind through a number of years until her application to me. Ordinary print, I found upon examination, she was not able to read at all, but by approximating large letters to within three inches of the eye, she was able to make them out. (This is explained by the fact that the indistinctness of the image does not increase in the same proportion as does its size from a near approximation of an object.) I found that with + 12, she was able to read No. 20 at twenty feet, but that for reading she required + 6, and with them was able to read and sew without any trouble and for any length of time. I instructed her to use the + 12 for distance, and the + 6 for near work, and since then have heard no complaint. In this case we have Presbyopia affecting a Hypermetrope, but of this we shall see more further on.

*ORIGIN.*

H. is nearly always congenital, and is very frequently hereditary. You very seldom see an isolated case of H. in a family. In these congenital cases it undoubtedly depends upon an arrest of development. Even the external appearance of an eye marks it as hypermetropic. It is often perceptibly smaller than the normal eye, and has a flatter look—which, however, does not come from a flattening of the cornea, but from the disproportion between the lateral and antero-posterior diameters of the eye. The greater curvature at the equator can be seen if the eye is turned strongly inwards. The physiognomy of the Hypermetrope is also peculiar and marked in many instances. The orbital edges are flatter than normal, giving a dished appearance to the face, and the anterior chamber is much shallower than in the emmotropic eye. There is also an *apparent* divergent strabismus. When an object at an infinite distance is fixed, the eyes assume a slightly diverging position. This is caused by the change in the angle made by the visual and optic axes. In the normal eye, the visual axis cuts the cornea *inside* of the optic axis, making an angle of about 5°. In M., it may sometimes cut the cornea *outside* of the optic axis, and thus produce an apparent squint *inward.* In H., however, it cuts the cornea to the inner side of the optic axis, and makes a greater angle than the normal, amounting sometimes to 11°. This is explained by two facts—first, the more outward position of the yellow apot; and second, the shortened antero-posterior diameter of the eye.

M. we found to be most common in the higher classes of society. On the contrary, H. is most frequently found in the lower ranks. It has been supposed by some, from this, that the different modes of life in the two classes may have some influence in the production of the different conditions. This has, however, not been satisfactorily proven.

*THE DETERMINATION OF H.*

is the same as that of M., viz.: by test-glasses and by the ophthalmoscope. In H., that positive glass which, during rest of A., brings parallel rays to a focus upon the retina, marks its degree. Thus, if with a + 12 a hypermetrope can read No. xx. at twenty feet, he has an H.=1-12; if it requires a + 6 to enable him to see the same accurately, the H.=1-6, and so on. But we must here avoid an error that our fathers in ophthalmology fell into: one that led to very unsatisfactory practice, and brought the treatment of the affection by glasses into disrepute. It is the error of overlooking any H. that may be *latent.* In all cases of *doubt,* especially in young persons, the A. should be paralyzed by atropine before the trial by glasses. Remember, also, it is the *strongest* glass with which distinct vision is possible that marks the degree of H., as in M. we saw it was the *weakest* that gave us its degree.

The determination by the ophthalmoscope is very convenient in the case of children who cannot read, or where a portion of the H. is suspected to be latent, and it is not desirable to paralyze A. In using the ophthalmoscope in the direct method, if there is any H., a distinct and well-defined view of the fundus is obtainable only with some Accommodation on the part of the observer. The amount of A. put forth by the observer gives a very good idea of the degree of H., but as this is not always determinable, it is best to use convex glasses behind the mirror and completely relax the A. The strongest glass, then, through which a well-defined view of the fundus is possible is the degree of H.

When the patient is old enough to have his A. interferred with by age, both his near and far point should be taken. The glass that gives a distinct image at distance in this case will not allow the patient to read in comfort. And even before the time when Emmetropes begin the use of glasses, the Hypermetrope will find himself compelled to increase the strength of his glasses, because his Hl. (latent Hypermetropia) is constantly giving place to Hm. For instance, if 1-12 gives good vision for distance, in a person of forty-eight years of age, you will find it necessary to give 1-6 for reading. Here we have H.=1-12, and Pr.=1-12. The second case given in this article is an instance of this.

### *COMPLICATIONS.*

The two most important of these are *Asthenopia* and *Strabismus.* It has been only quite recently that their intimate connection with H. has been clearly understood. Here, again, the world of science and suffering humanity owe a great debt of gratitude to Donders. Before this, the asthenopic was considered without the pale of therapeutics, and any degree of comfort was only obtainable by abstinence from work. But by showing its dependence upon H., Donders has provided us with a remedy as prompt as it is effective.

We shall first consider *Asthenopia*, which, as its name implies, is painful vision. In studying M., we found there a painful vision, but this has an origin in an entirely different portion of the eye from the asthenopia of H. In M., it depends upon the fatigue of the internal rectus muscle, while in H. it comes from fatigue of the ciliary muscle, or muscle of Accommodation. In H., we have seen, even distant objects are seen distinctly only by using a portion of the A.; and of course, as the object is approximated nearer the eyes, a still greater amount of A. is required for distinct vision. This extra amount and constant tension of the ciliary muscle must bring on fatigue, just as the muscles of the arm become tired if subjected to the constant strain of sustaining a continuous weight. In the Hypermetrope there is no rest of A.; to see at all distinctly, whether near or distant objects, requires an effort. But, of course, the nearer the object, the greater must be the effort put forth, and then it is when the feeling of fatigue manifests itself.

The symptoms of As. are well-marked. The patient finds it impossible to continue near work for any length of time without pain. The pain is different from the As. of M., in that it is referred to the brow and forehead, instead of to the eye itself. If work, in spite of these warnings, is still continued, objects become so confused and indistinct as finally to make it impossible. This indistinctness is the result of the giving way of the ciliary muscle under the excessive strain. After resting the eyes, however, for some time, work can be again resumed with a degree of comfort, but if it is continued, the same symptoms again manifest themselves, necessitating another suspension of work. In mild cases, the only symptom that may be noticed is an aching in forehead after a rather prolonged use of the eyes, especially in artificial light. This complication is most frequently met with in the *Facultative* or lowest form of H. This might at first sight seem strange, but upon consideration we can readily see the cause. In Absolute H., no amount of A. can make near vision acute. The patient soon learns this, and chooses rather to submit to the indistinctness than strain his A., especially as this will do him but little good. For the same reason, it is most generally absent in Relative H., for, in order to obtain distinct vision, there must be a degree of squint, which would, of of course, produce double images; hence, rather than give up binocular vision, an indistinctness is tolerated, and a large amount of extra A. is not called for. But when, by an extra effort of the A., binocular vision *is* possible, this extra amount will be called into requisition, with a consequent strain of the ciliary muscle. From this strain As. arises.

The second important complication of H. is *Strabismus*, and as a law, of the convergent variety. The subject of squint itself would take, for its proper consideration, more space than is occupied by this entire article, and we can do no more here than simply touch upon its production by H.

Strabismus from H. depends upon the close relationship existing between the action of the internal recti and ciliary muscles, considered in the first part of this article.

The manner of its production is as follows: The extra A., as we have seen, that is required for distinct vision at a specified distance—say eighteen inches—brings into play (from associated action) a convergence that will bring the binocular nearest point (the crossing of the visual axes) nearer the eyes, say fourteen inches; fixing and accommodating for the same point is thus rendered impossible. But if one eye is allowed to deviate inwards under the associated action of the rectus muscle, accommodation *with one eye* for that point (eighteen inches) is possible. And this is what is most commonly done in the Relative form of H. We have, then, in this form, binocular vision impossible. But as squinters very seldom complain of double vision, what becomes of the image in the deviating eye? It is *suppressed*. This suppression, which is called *active* suppression, is a purely psychical act, and, if continued for some time, the capacity for vision in that eye becomes very seriously impaired. By use, however, this power can in a great measure be regained.

*THE TREATMENT*

of H. is simple, and in the majority of cases eminently satisfactory. It consists in the use of convex glasses of such a focal length as will bring parallel rays to a focus upon the retina during rest of

A. Distinct vision with the normal amount of A. is thus made possible; the strain upon the ciliary muscle is taken away, and the asthenopic pain disappears as if by magic. The relation between the A. and convergence is restored to its normal condition, and the tendency to squint at once arrested. What greater triumph in the healing art can there be than this? All children who show a tendency to squint, should be examined for H., and if it be found and corrected, the squint is prevented, and the unsightliness caused by it, and the operation for its cure in later life, are avoided; for, if the squint is allowed to develop, that which was in the beginning simply functional and susceptible of relief by glasses, becomes in time organic, and can only be cured by an operation.

Theoretically, it would seem proper to neutralize the total amount of H., and then your patient would be on a par with Emmetropes. But practically, this will not answer in all cases. The A. in most instances has in a degree accommodated itself to the abnormal relation, and it is best to first neutralize only the Hm., and gradually increase the strength of the glasses until the Ht. is neutralized.

As the A. begins to give way with age, you will have, of course, to correct the Presbyopia just as you would in the Emmetrope. In these cases, the ordinary glasses will be used for distance, and stronger ones for near work, whose strength will have to be increased with advancing years.

There is a popular prejudice against the early use of glasses, than which nothing can be more pernicious. We have seen in the foregoing that Squint and Asthenopia can be prevented by the proper use of glasses, and when these symptoms make their appearance, no matter at what age, means should be taken for their relief, as the results of their neglect are often disastrous.

---

Tears in Children.—M. Trousseau states, as a general rule, that when the infant sheds tears it is not dangerously ill; and, on the contrary, the absence of weeping indicates a severe disease. He regards this to be so true as to deserve to be considered as an aphorism. He does not deny, however, that there may be exceptions to this general rule.

